# Pain Behavioural Response to Acoustic and Light Environmental Changes in Very Preterm Infants

**DOI:** 10.3390/children8121081

**Published:** 2021-11-24

**Authors:** Audrey Marchal, Meggane Melchior, André Dufour, Pierrick Poisbeau, Claire Zores, Pierre Kuhn

**Affiliations:** 1 Service de Médecine et Réanimation du Nouveau-né, Hôpital de Hautepierre, Centre Hospitalier Universitaire de Strasbourg, 67000 Strasbourg, France; audrey.marchal1987@gmail.com (A.M.); claire.zores@chru-strasbourg.fr (C.Z.); 2 Institut des Neurosciences Cellulaires et Intégratives (INCI, CNRS UPR-3212), Centre National de la Recherche Scientifique, 67000 Strasbourg, France; mmelchior@unistra.fr (M.M.); poisbeau@inci-cnrs.unistra.fr (P.P.); 3 Laboratoire de Neurosciences Cognitives et Adaptatives (LNCA, CNRS UMR-7364), Centre National de la Recherche Scientifique, Université de Strasbourg, 67000 Strasbourg, France; andre.dufour@unistra.fr

**Keywords:** acute pain behaviour, preterm infants, environmental stressors

## Abstract

Noise and high light illumination in the neonatal intensive care unit (NICU) are recognized as stressors that could alter the well-being and development of vulnerable preterm infants. This prospective observational study evaluated the pain behaviours of very preterm infants (VPIs) to sound peaks (SPs) and light levels variations (LLVs) in the NICU. We measured spontaneously occurring SPs and LLVs in the incubators of 26 VPIs over 10 h. Their behavioural responses were analysed through video recordings using the “Douleur Aigue du Nouveau-né” (DAN) scale. We compared the maximum DAN scores before and after environmental stimuli and the percentage of VPIs with a score ≥ 3 according to the type of stimuli. A total of 591 SPs and 278 LLVs were analysed. SPs of 5 to 15 dBA and LLVs significantly increased the maximum DAN scores compared to baseline. The occurrence of DAN scores ≥ 3 increased with both stressors, with a total of 16% of SPs and 8% of LLVs leading to quantifiable pain behaviour. Altogether, this study shows that VPIs are sensitive to SPs and LLVs, with a slighter higher sensitivity to SPs. The mechanisms leading to pain behaviours induced by noise and light changes should be evaluated further in the context of VPIs brain development. Our results provide further arguments to optimize the NICU sensory environment of neonatal units and to adapt it to the expectations and sensory abilities of VPIs.

## 1. Introduction

Despite recent advances in neonatal care, about 40% of very preterm infants (VPIs), born before 32 weeks of gestational age, have neurodevelopmental sequelae in France and in high income countries [1,2,3,4]. Among the possible causes of disability in this vulnerable population are inappropriate stimuli or traumatic events during critical periods of brain development. A very early preterm birth exposes the newborn infant to abundant and atypical sources of stimulation [5], inducing stress-mediated mechanisms that may lead to neurosensory impairments. VPIs are also exposed to numerous and repeated painful stimuli [6,7], which have been shown to have consequences later in life [8,9]. In particular, painful stimuli during caring procedures have been shown to affect brain growth and functions in VPIs [9,10,11]. A greater exposure to invasive procedures also seems to contribute to lower intelligence quotients at school age [12]. The sensory systems of the newborn infants, although still immature at birth, are among the first to develop and are functional during the last trimester of pregnancy, and VPIs are well equipped to react to the neonatal intensive care unit (NICU) environment [4,5]. 

VPIs hospitalized in NICUs are highly exposed to a variety of environmental stressors, including auditory and light stimuli [13,14,15]. The light and noise conditions in NICUs contribute to the instability of VPIs and can in the long-term lead to an alteration of their neurosensory development [13]. Noise has short-term deleterious physiological effects on VPIs, especially on autonomous function [16,17]. VPIs react to moderate variations in illumination in their incubator [18], which tend to impair their physiological well-being and disrupt their sleep [18,19], leading to a potential alteration of the development of circadian rhythms [20,21]. Thus, in view of these already known detrimental impacts, it seems legitimate to question the possible effects of auditory and light stimulation on VPIs’ behavioural well-being and whether they could lead to discomfort or pain. 

This prospective observational study aimed to assess whether the spontaneous auditory and luminous stimulations occurring in the incubators of VPIs can be a source of discomfort, measurable through a pain scale. The secondary objectives were to evaluate the main determinants of these pain behavioural responses to auditory and luminous stimulations by studying the impact of the sleep state and of the intensity of the stressors.

## 2. Materials and Methods

The study cohort consisted of 26 VPIs hospitalized between April 2008 and July 2009 in the NICU of Strasbourg University Hospital, France. Infants with major congenital anomalies, significant intraventricular haemorrhages (grade > II) and/or parenchymal brain injuries and those treated with analgesics or sedatives during the previous 48 h were excluded. 

All infants were cared for in Draeger SC incubators (Draeger Medical, Lubeck, Germany) in rooms containing three or four beds. Infants were examined during a 10-h daytime period while lying in the prone position on their back or sides. Basic developmental care measures were used, including provision of skin-to-skin contact with parents, incubator coverings, nesting and general measures for sound abatement, but there was no specific program for noise reduction. All eligible infants were included on a convenience inclusion basis after parental information and consent.

### 2.1. Procedures 

#### 2.1.1. Environmental Measurements

Sound pressure levels and environmental sounds were recorded using a noise dosimeter (MS 6701-PRO Digital sound-level meter; Mastech, Pittsburgh, PA, USA) in A-weighted scale-slow response. A microphone was placed in the incubator, 5 cm above the blanket. None of the probes were in contact with the mattress at any time. Equivalent sound levels were recorded at 1-sec intervals.

Regarding illumination, daylight could enter through windows with manual flaps to protect occupants from direct sunlight. The incubators were parallel to the windows. Artificial light sources were ceiling-modulated neon lights and individual mobile-focused lamps. Illumination was measured using a lux meter, a digital light meter RS180-7133 (RS Components, Northants, UK) placed at the infants’ eye level. The 5–10 cm distance between the eyes and the lux meter, and their similar orientation, allowed a similar incidence of illumination to reach the lux meter and the newborn infants’ eyes. Light measurements were recorded in lux (lm/m^2^) at two-sec intervals.

#### 2.1.2. Behavioural Data Collection

Two cameras were placed outside each incubator and used to video record each infant during the observation period. Body movements and facial expressions were analysed. Arousal states were assessed using Prechtl’s observational rating system by two specifically trained observers unaware of the study’s purpose [22]. The examiners were allowed to watch the videotape as they needed. In the case of conflicts in the initial scoring, the examiners jointly re-evaluated these periods to reach a final agreement. Periods of infant care, sources of multi-sensory stimulations and periods of skin-to-skin contacts (i.e., outside the incubator) were excluded from the analysis.

#### 2.1.3. Data Analysis

As already described [17,18,19,23], to evaluate only the infant’s responsiveness to noise and light in the incubator only the behavioural data in quiet sleep or active sleep of the Prechtl classification [22] were retained. We excluded periods of direct skin contact during routine care or when the incubator door was opened and periods following respiratory support changes. 

#### 2.1.4. Acoustic Environment

Sound peaks (SPs) were defined as a 1-sec sound pressure level (equivalent continuous level) exceeding the previous 1-sec interval by 5 dBA equivalent continuous level or more, classified in 5 dBA equivalent continuous level increments and reported as signal-to-noise ratio (SNR) in dBA. Thus, the reference for SP measurements was the background sound pressure levels measured in dBA for each SP. Subgroups were identified for SNR ranges 5–10 dBA and 10–15 dBA and by baseline sleep state (active sleep and quiet sleep) preceding the occurrence of each SP. These thresholds were determined since they correspond to the auditory perceptual abilities of VPI, as previously described [17]. 

#### 2.1.5. Light Environment

Changes in lux levels were identified as a deviation above the previous 2-sec interval and classified into two ranges: variations of 10 to 50 lux and of more than 50 lux. Non-isolated light level variations (LLVs) were excluded from the analysis. Subgroups were identified for LLV of 10 to 50 lux and LLV > 50 lux and by baseline sleep state (active sleep and quiet sleep) preceding the occurrence of each light peak. The threshold of 10 lux was chosen since it corresponds to the visual perceptual abilities of VPI, as previously described [19].

#### 2.1.6. Behavioural Data Analysis

Behaviour was evaluated starting 10 sec before each SP or LLV and for the following 40 s in 10 s increments, using the “Douleur Aigue du Nouveau-né” (DAN) scale. This multidimensional behavioural scale designed to assess procedural pain in preterm and full-term infants has a good internal consistency and interrater reliability [24] and was shown to have strong concurrent validity with the widely used and validated Premature Infant Pain Profile Scale [25,26]. 

We considered two periods: baseline, corresponding to the 10 s preceding a change in illumination/sound levels, and post stimulation, corresponding to the 40 s following the change in light or sound levels. The exact times of each 10-s period were indicated to the blinded observers.

To evaluate behavioural changes, we compared the baseline DAN score to the maximum DAN score of the four periods of 10 s following SPs/LLV. The number of DAN scores ≥ 3 and number of VPI presenting DAN scores ≥ 3 were also analysed. This is considered a threshold to provide the newborn with pain treatments as the DAN score was shown to differentiate painful from non-painful procedures with a score > 3 in 95% of painful procedures and <2 in 88% of dummy procedures [24].

### 2.2. Statistical Analysis

We first realised a descriptive analysis of the SPs and LLV according to the sleep states and the intensity of the stimulation. Then, for each VPI, the means of baseline DAN scores and maximum post-stimulation DAN score were calculated. To evaluate the impact of sound and light variations on VPIs’ behaviour, we compared the DAN score between the baseline period and the post-stimulation periods using a Wilcoxon matched-pairs signed rank test. To analyse the impact of sleep state and of the intensity of the stimulus, we used a two-way analysis of variance (ANOVA). The distribution of the number of DAN scores ≥ 3 according to the SNR ranges and the sleep states was analysed using the Pearson χ^2^ test. To analyse the proportion of VPIs with a DAN score ≥ 3 according to the SNR ranges and the sleep states, we had to use the Fisher’s exact test due to the small number of subjects. Finally, to compare the effect of SPs and LLVs, the magnitude of variation (i.e., difference of maximum post stimulation DAN scores—baseline DAN score) was calculated. 

Post hoc analyses were performed, when appropriate, using the Newman–Keuls test. Differences with *p* < 0.05 were considered statistically significant. The number of SPs and LLVs per child are shown as median [range] and all other results are shown as mean ± standard deviation (SD).

## 3. Results

### 3.1. Study Population

The characteristics of the study population are presented in Table 1. Among this population, 22 VPIs had exposure to both auditory and visual light stressors.

### 3.2. Changes in the Acoustic Environment

Only SPs occurring during active sleep or quiet sleep states and between 5 to 15 dB above background sound levels were analysed, leading to a total of 591 SPs and corresponding to a median number of 20 [2–71] per child. The vast majority of them were of low SNR between 5 and 10 dBA (514 SPs, 87%). Only 77 (13%) of recorded SPs had a SNR between 10 and 15 dBA. A total of 201 (34%) SPs occurred in quiet sleep and 390 (66%) SPs in active sleep. Table 2 shows the distribution of the number of SPs per child according to the categories of SNR and sleep states at the time of their occurrence.

### 3.3. Changes in Environmental Light Levels

Only LLVs occurring during quiet sleep and active sleep states were analysed, leading to a total of 278 LLV, corresponding to a median number of 12 [1–38] LLVs per child. The majority of them varied between 10 and 50 lux (222 LLVs, 80%). Only 56 (20%) LLVs were higher than 50 lux. A total of 256 (92%) LLVs occurred in quiet sleep and 22 (8%) in active sleep. The number of LLVs per child, related to the sleep state and the intensity of light variations, are shown in Table 2.

### 3.4. Pain Behaviour and Environmental Changes

#### 3.4.1. Acoustic Changes and Pain Behaviour

**Figure 1:**
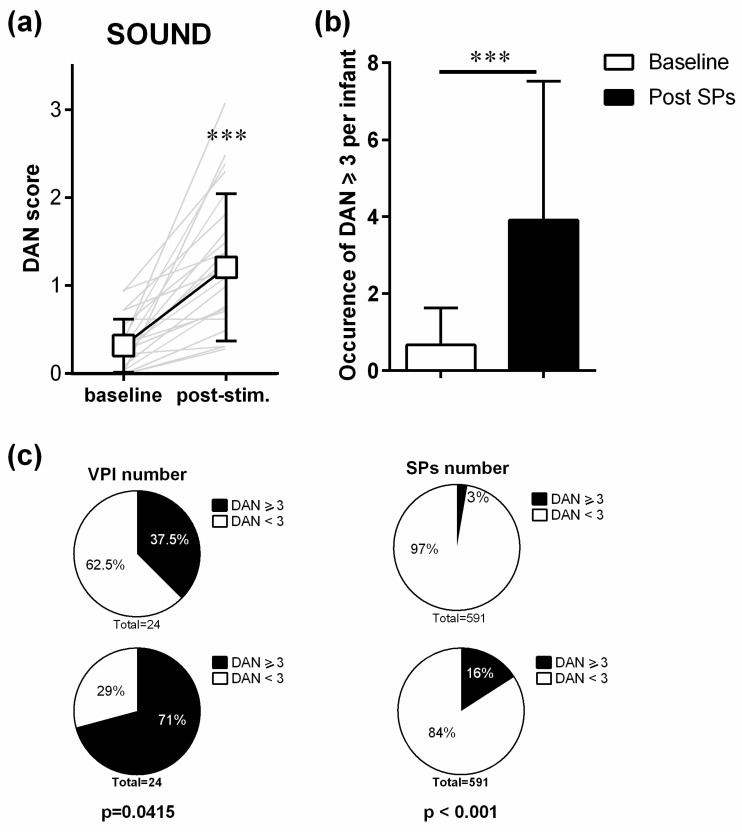
Impact of sound peaks on pain behaviours. (**a**). Mean (±SD) DAN scores before (baseline) and after (maximum) sound stimulations (**b**). Number of DAN scores ≥ 3 detected per VPI at baseline and after sound peak. (**c**). Proportion of VPIs and SPs number in the different DAN categories, at baseline (upper panel) and after SPs (lower panel). Grey lines represent the individual variations for each infant. *** *p* < 0.001 as compared to baseline (Wilcoxon matched-pairs signed rank test, *n* = 24). In panel c, *p* values are indicated for Fisher exact test and Pearson test for VPI and SP numbers, respectively. SPs = sound peaks, VPIs = very preterm infants.

Behavioural assessment of VPIs after all SPs showed a significant increase in maximal DAN scores (Wilcoxon matched-pairs signed rank test, W = 231, *p* < 0.001; *n* = 24), with an average baseline DAN score of 0.32 (±0.3) and of 1.21 (±0.8) after stimulation (Figure 1a).

To determine whether SPs lead to pain behaviour according to the standard clinical criteria in VPIs, we evaluated the occurrence of DAN scores ≥ 3 for all SPs. SPs induced an increased occurrence of DAN scores ≥ 3 (Figure 1b; Wilcoxon matched-pairs signed rank test, W = 167, *p* < 0.001; *n* = 24). At baseline during the 10 sec of evaluation, DAN scores ≥ 3 were detected only 16 times (3%), in 9 out of 24 VPIs (Figure 1c). Then, of the 591 SPs analysed, 94 (16%) led to quantifiable pain behaviour with a DAN score ≥ 3, which concerned 17 out of 24 VPIs (Figure 1c). Comparison of these frequencies confirmed that there was a significant higher amount of pain behaviour (i.e., number of DAN scores ≥ 3) after SPs (VPI: Fisher exact test *p* = 0.0415; SPs: Pearson χ^2^ test, Chi-square = 60.98; df = 1; z = 7.809, *p* < 0.001). The highest DAN scores that were observed reached 9 out of 10 (in active sleep and for SPs between 5 and 10 dBA). Two VPIs reached this maximum.

#### 3.4.2. Determinants of Behavioural Change

Behavioural changes following SPs were analysed according to SNR intensity and sleep state. The comparison of baseline and post SPs DAN scores showed that SPs increased the DAN score when the child was in active sleep for both categories of intensity and when the child was in quiet sleep but only for SPs of 5–10 dBA (Wilcoxon matched paired rank test). These results are presented in Table 3.

When comparing the variations of DAN scores (delta DAN score), we observed no effect of either the sleep state or the intensity of the SPs (two-way ANOVA; intensity * sleep state; *p* = 0.14).

Then, we analysed the distribution of the number of DAN scores ≥ 3 according to the SNR ranges and the sleep states. Pain behaviours were mainly found in VPIs in active sleep: 17.5% (58/331) of the SPs of 5 to 10 dBA SNR and nearly a third (18/59) of the SPs of 10 to 15 dBA SNR triggered pain behaviour. In quiet sleep, we observed less pain behaviour in response to SPs with 8.7% (16/183) and 11.1% (2/18) of DAN scores ≥ 3, respectively for SPs of 5–10 dBA and 10–15 dBA. Comparison of these frequencies using a Pearson χ^2^ test showed a significantly greater amount of pain behaviour in response to SPs in active sleep, whatever the SNR intensity (76/390 or 19.5% of DAN scores ≥ 3 for SPs in active sleep vs. 18/201 or 9% of DAN scores ≥ 3 for SPs in quiet sleep, Chi-square = 11; df = 1; z = 3.32; *p* < 0.001). Similar results were obtained when comparing the number of VPIs reporting DAN scores ≥ 3 (11/26 or 42% of VPIs in active sleep vs. 3/26 or 12% in quiet sleep; Fischer exact test, *p* = 0.0266). 

The intensity of the SP also impacted the pain behaviour with significantly less DAN scores ≥ 3 in response to SPs of 5 to 10 dBA SNR (74/514 or 14%) than to SPs of 10 to 15 dBA SNR (20/77 or 26%) (Chi-square = 6.7; df = 1; z = 2.59; *p* = 0.0096). However, when comparing the number of VPIs reporting DAN scores ≥ 3 the difference between the SPs between 5 and 10 dBA SNR (10/26 or 38%) and SPs between 10 to 15 dBA SNR (4/26 or 15%) was not significant (Fischer exact test, *p* = 0.1164).

#### 3.4.3. Variation in Light Levels and Pain Behaviour

**Figure 2:**
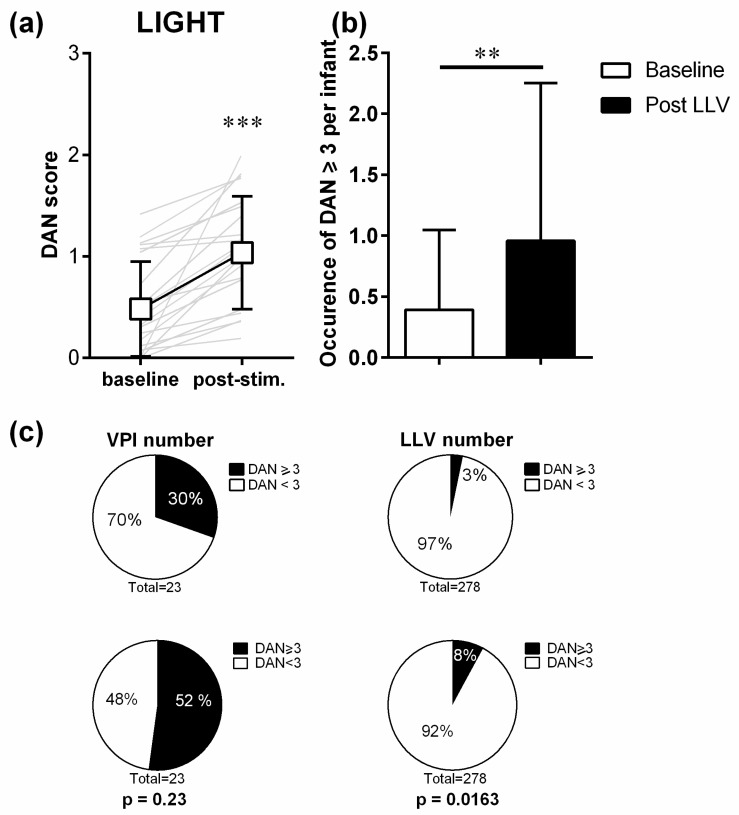
Impact of light level variations on pain behaviours. (**a**) Mean (±SD) DAN scores before (baseline) and after (maximum) light stimulations. (**b**) Number of DAN scores ≥ 3 detected per VPI at baseline and after LLV. (**c**) Proportion of VPIs and LLVs number in the different DAN categories, at baseline (upper panel) and after LLVs (lower panel). Grey lines represent the individual variations for each infant. *** *p* < 0.001 ** *p* < 0.01 as compared to baseline (Wilcoxon matched-pairs signed rank test, *n* = 23). In panel c, *p* values are indicated for Fisher exact test and Pearson test for VPI and LLVs numbers, respectively. LLV = light level variation, VPI = very preterm infants.

We observed a significant increase in maximal DAN scores, whatever the light level changes and the sleep state (Wilcoxon matched-pairs signed rank test, W = 253, *p* < 0.001; *n* = 23), from an average baseline DAN score of 0.48 (±0.47) to 1.04 (±0.56) after LLV (Figure 2).

LLVs induced an increased occurrence of DAN scores ≥ 3 (Figure 2b; Wilcoxon matched-pairs signed rank test, W = 45, *p* = 0.0039; *n* = 23). At baseline during the 10 s of evaluation, DAN scores ≥ 3 were detected only 9 times (3%), in 7 out of 23 VPIs (Figure 2c). Then, of the 278 LLVs analysed, 22 (8%) lead to quantifiable pain behaviour with a DAN score ≥ 3, which concerned 12 out of 23 VPIs (Figure 2c). Comparison of these frequencies confirmed that there was a significant higher amount of pain behaviour (i.e., number of DAN scores ≥ 3) after LLVs, when considering LLVs numbers but not when considering VPIs numbers (VPI: Fisher test *p* = 0.2307; SPs: Pearson χ^2^ test, Chi-square = 5.774; df = 1; z = 2.403, *p* = 0.0163). The highest DAN scores that were observed reached 3 out of 10. 

#### 3.4.4. Determinants of Behavioural Changes

Behavioural changes following LLV were analysed according to light level and sleep state. The comparison of baseline and post LLV DAN scores showed that LLV increased the DAN score when the child was in quiet sleep for both categories of intensity and when the child was in active sleep for LLVs between 10 to 50 lux (Wilcoxon matched-pairs rank test). As we recorded only one LLV of more than 50 lux in active sleep, no specific evaluation was possible in this subgroup. These results are presented in Table 4.

Then, we analysed the distribution of the number of DAN scores ≥ 3 according to the LLV intensity and the sleep state. In quiet sleep, 8% (16/201) of the LLVs of 10 to 50 lux and nearly 7.3% (4/55) of the LLVs higher than 50 lux triggered pain behaviour. In active sleep, 4.8% (1/21) of LLVs between 10 to 50 lux induced DAN scores ≥ 3. Comparison of these frequencies using Pearson χ^2^ tests showed no significant differences in the proportion of LLVs leading to pain behaviour. Similarly, no difference in the proportion of VPIs with pain behaviour in the different categories was observed using the Fischer exact test. In quiet sleep, 43% (9/21) of VPIs had pain behaviour in response to 10–50 lux LLVs and 18% (3/17) of VPIs in response to LLVs higher than 50 lux. In active sleep, 10% (1/10) of VPIs had pain behaviour in response to 10–50 lux LLVs.

#### 3.4.5. Comparison of Pain Behaviours Triggered by Sound and Light Changes

Overall, the impact of SPs seems stronger than the impact of LLVs, since when comparing the proportion of peaks inducing DAN scores ≥ 3, we observed a significantly higher proportion for SPs than for LLVs (Chi-square = 10.44; df = 1; z = 3.231; *p* = 0.0012). However, the proportion of VPIs with peaks leading to DAN scores ≥ 3 was similar for SPs and LLVs (Fischer exact test; *p* = 0.1205). Moreover, the highest DAN score observed was higher with SPs (9 out of 10) than with LLVs (3 out of 10).

**Figure 3:**
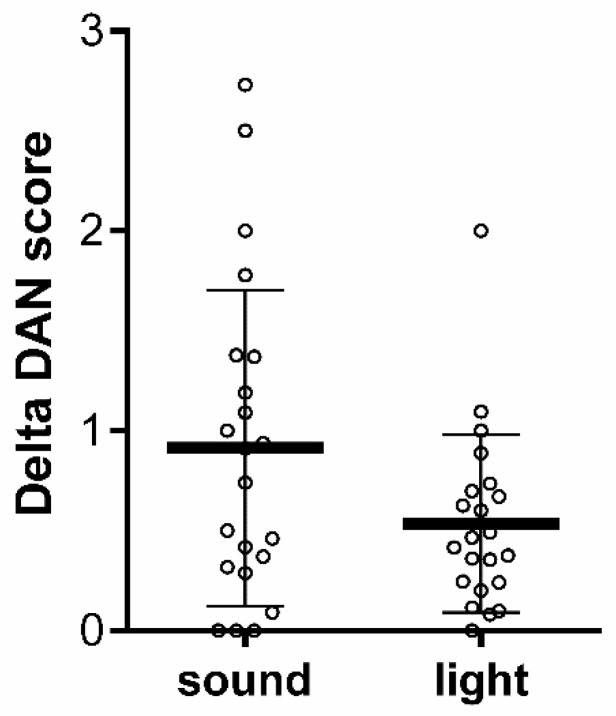
Means of the maximum significant changes of the DAN score as compared with baseline during the 40 s following SPs and LLVs for all sleep states and SNR intensities.

Then, to compare the changes in the mean of the DAN scores after SPs and LLVs, only the VPIs who had both SPs and LLVs were included in the analysis in order to avoid variability in the data and to perform a statistical analysis with repeated measures. The comparison showed a tendency of smaller variations in response to LLVs, but it did not reach statistical significance, with a mean of 0.91 ± 0.79 for SPs and 0.53 ± 0.44 for LLVs (Wilcoxon matched-pairs rank test; *n* = 22; W = -105, *p* = 0.0701) (Figure 3).

## 4. Discussion

In this study, we evaluated the behavioural responses of VPIs to sound and light variations during their hospital stay. We found that spontaneous environmental SPs and LLVs, at levels already reported in the literature [14], induced significant behavioural changes measured as an increase in DAN score, a scale classically used to assess acute pain in VPIs. The proportion of VPIs and SPs or LLVs with DAN scores ≥ 3 also increased after the stimulations. Altogether, our results suggest that SPs have a stronger impact than LLVs on pain behaviour in VPIs. 

This evaluation of behavioural changes in response to SPs and LLVs provides a better understanding of the responses of VPIs to their “natural” sound and light environment in NICUs. It has been described that VPIs are subjected to a median of approximately 7 SPs higher than 5 dBA per hour, mainly above 15 dBA, with fundamental frequencies ranging from 100 (incubator’s motor) to 2730 Hz (syringe pump alarm) [17]. Concerning LLVs, we previously described a median of approximately 3 isolated LLVs per hour, with a majority of LLVs being between 10 and 50 lux [18]. Our team previously demonstrated that the auditory and visual perceptual abilities of VPIs allow them to detect low sound variations with SNRs between 5 and 15 dBA and low LLVs of at least 10 lux. These moderate environmental changes are known to significantly alter their physiological well-being and disrupt their sleep [19,23]. Noise indeed decreased oxygen saturation and increased their heart rate [17]. Moderate light changes between 10 and 50 lux significantly increased their respiratory rate [18], and increases in heart rate and decreases in oxygen saturation and cerebral oxygenation were observed in response to more intense light variations (>50 lux). Our data add to the possible burden of moderate light/sound changes on the immediate well-being of VPIs. 

We performed some sub-analyses based on the number of VPIs or stimulations leading to a DAN score ≥ 3, as this is considered a threshold to provide the newborn with pain treatments. In this study, 16 and 8% of analysed SPs and LLVs, respectively, led to a DAN score ≥ 3. This corresponded to 71 and 52% of VPIs with a DAN score ≥ 3 in response to SPs or LLVs, respectively. The intensity of the SPs, but not LLVs, and the sleep state impacted the pain behaviour, with more DAN scores ≥ 3 in the active sleep state. However, the number of LLVs higher than 50 lux was limited in this study, as only one was observed in the active sleep state. Similarly, the number of SPs of 10–15 dBA observed per infant in QS is quite low (median 0.5 and range from 0 to 3) compared to SPs of 5–10 dBA (median 4 and range from 0 to 42), which could explain why we did not observe a significant increase in maximum DAN score after SPs of 10–15 dBA but only after SPs of 5–10 dBA. These observations correlate well with the previous data demonstrating a higher autonomic reactivity of newborn in active sleep state [27,28,29].

However, as for numerous non-verbalizing patients, these results raise the question of whether the observed behavioural changes were indeed associated with a real pain experience. There is no doubt anymore that VPIs are sensitive to pain. Cortical integration of pain has been demonstrated in newborns at a very early stage of life [30,31,32,33,34,35,36]. In VPIs, discriminative and specific pain responses to noxious stimuli have been detected as soon as 33 weeks of GA [37,38]. However, assessing pain in newborn infants and being able to distinguish it from “stress” using behavioural cues remains a challenge. To date, there is no consensus on a definition allowing the distinction between “pain” and “stress” in neonatology. The team of Carbajal et al. defined neonatal pain as “any event that violates the integrity of the newborn’s body, causing skin or mucous damage through the introduction or removal of foreign material from the airways, digestive tract or urinary tract” [6]. A stressful procedure was defined as any event causing stress or discontent or disrupting the balance between the newborn and his environment. Thus, behavioural responsiveness in response to sound and light stimuli appears certainly to trigger rather stress-like responses and discomfort than real pain, if the criteria set by Carbajal are considered. In our study, the VPIs’ age ranged from 28 to 34 weeks, when the discriminative response to noxious stimuli is not observed yet [37]. However, at this stage, it is not possible to exclude that these stimuli induce pain as most of the pain messages are transmitted by non-noxious A-fibers during early stages of development [39]. 

The DAN scale or other widely used and validated observational scales are based on a hetero-evaluation of the pain. They are exposed to the risk of observation bias and do not allow continuous monitoring of pain responses. Continuous monitoring techniques have been developed to address some of these deficiencies and are based on the analysis of variations in autonomic parameters of the child: skin conductance [40] and heart rate variability [41]. Unfortunately, these tools also have limitations and measure parameters which are not specific for pain. Their use could, however, allow the discomfort associated with sound or light stimulation originating from the hospital environment of the newborns to be detected, as it was demonstrated in response to sound exposure [42]. Exploring the cortical activations in brain areas integrating pain might allow us to decipher whether or not the behavioural response to SPs and LLVs triggers real pain, as previously shown in VPIs in response to nosocomial odour exposure using behavioural and NIRS assessment [43]. However, very little work has been done to combine these different indicators or to compare them with cortical responses, which are the only ones to reflect a “conscious perception” of pain. A multimodal approach is therefore essential to better assess the pain of the newborn and its possible presence in response to environmental stimulation.

These data also raise the question of the possible deleterious short- and long-term consequences of repeated sound and light stimulations. It is indeed well accepted that the high exposition of VPIs to nociceptive and stressful stimuli during care [6] may impair their brain development [44], leading to decreased cortical thickness [9], slower development of somatosensory regions [45], poor cognitive function [12,46] or altered pain sensitivity [47]. Animal models also emphasized the impact of the early sensory environment, including pain, stress and maternal contact, showing cognitive and social alterations and altered responses to stress and pain at adulthood in animals subjected to early life stress [48,49]. However, the specific long-term effects of sound and light exposure have not been investigated yet in this context (i.e., for stimulations in this range of intensity). However, the deleterious effects of brief and high sound pressure levels (greater than 135 dBA during an acoustic shock) or a prolonged exposure to intense noise (greater than 80 dBA for more than 8 h) are well known in the adult and paediatric population. These may trigger pain and a major and irreversible hearing loss [50]. Only a few studies have evaluated the painful impact of an intense exposure to noise and mainly focused on people with hyperacusis [51,52], who experience severe pain and in the ear/or head [51]. In children, hyperacusis may be associated with genetic disorders [53] and behavioural disorders [54] but may also occur because of single or repeated sound trauma [55]. Despite the fact that hyperacusis was described for SPs much higher than the those observed in this study, we cannot exclude that VPIs exposed to many environmental auditory stimuli during their sensory development would therefore be potentially at risk for hyperacusis. Yet, there is currently no study of the incidence of hyperacusis in the population of former VPIs. 

In any cases, all NICUs should establish a pain prevention program, including pharmacological and non-pharmacological treatment to prevent pain induced by minor or major procedures [56]. However, stressful situations should not require medical analgesic treatment, especially morphine, which can be deleterious in the short and long term for newborns [57]. In view of very high DAN scores (9/10) observed in response to SPs, preventive management is still necessary, to limit the short-term and long-term consequences of these repeated stressful stimulations [9,12,58,59]. Given the short-term adverse behavioural effects of noise and light that we have shown and its possible long-term repercussions, it seems fully justified that each NICU strives to minimize the exposure of VPIs to excessive or inappropriate environmental stimulation. These efforts are an integral part of the infant and family centred developmental care strategies and programs supported at national [60,61] and European levels [62]. Sound pressures and light exposure should not be considered solely in terms of absolute intensity values but also in terms of numbers of hourly and daily peaks and SNR or light variations. Pioneering studies on the physiological and behavioural responses to different stimuli considered painful in NICUs [63] have served as a basis for the clinical evaluation scales in this area, leading to increased awareness and better management of neonatal pain [64].

## 5. Conclusions

Our study evaluated the impact of the hospital environment on the behaviour of VPIs in response to repeated sound and light stimulations. The results of this study highlighted the discomfort triggered by sound and light stimuli of various intensity. Although DAN scores following these stimuli sometimes reached values considered to be painful, it is now necessary to investigate whether VPI discomfort is indeed pain and leads to processing in central pain related structures. More generally, the connections between multi-sensory perception and nociception deserve to be better assessed especially in the population of VPIs where very little data is available. Our study could therefore have extensions in clinical but also in fundamental research. However, in addition to the basic scientific knowledge provided by this work, our results are already a support to optimize the clinical environment of the NICU and to adapt it to the needs and sensory abilities of VPIs. Ultimately, this should help to improve their subsequent neurodevelopment as part of developmental care strategies. 

## Figures and Tables

**Table 1:** Main characteristics of the study population.

GA, median weeks [range]	28 [26–31]
Birthweight, mean gram (SD)	1109 (±250)
Post-natal median age in days [range]	17 [4–50]
Postmenstrual age, median days [range]	31 [28–34]
Gender (girls-boys; *n*)	10–16
Small for GA/Adequate for GA (*n*)	8–18
Respiratory support at time of study (*n*)	
Room air	9
nCPAP ^a^	9
Mechanical ventilation ^b^	8
Duration of respiratory support, median days [range]	
Oxygen supplementation	16 [0–36]
nCPAP ^a^	6 [0–30]
Mechanical ventilation ^b^	3 [0–29]

^a^ nCPAP nasal continuous positive airway pressure (nCPAP, Infant Flow Driver, Sebac, Genevilliers, France). ^b^ Mechanical ventilation (MV-Babylog 8000, Draeger Medical, Lübeck, Germany). GA = gestational age; SD = standard deviation.

**Table 2:** Number of SPs and LLVs per child according to the sleep state and the intensity of sound/light variation.

	QS Median [Range]	AS Median [Range]
SPs 5–10 dBA	4 [0–42]	12 [0–48]
SPs 10–15 dBA	0.5 [0–3]	1 [0–19]
LLVs 10–50 lux	7 [0–32]	0 [0–5]
LLVs > 50 lux	1 [0–9]	0 [0–1]

QS = quiet sleep, AS = active sleep. Sound pressure levels are expressed in dBA.

**Table 3:** DAN score changes according to SNR intensity and sleep states.

		DAN Score Baseline (Mean ± SD)	Maximum DAN Score Post Stimulation (Mean ± SD)	N; *p* Value
QS	5–10 dBA	0.26 ± 0.69	1.32 ± 1.73	19; <0.001
	10–15 dBA	0 ± 0	0.57 ± 1.18	12; 0.125
AS	5–10 dBA	0.44 ± 0.46	1.19 ± 0.76	23; <0.001
	10–15 dBA	0.55 ± 0.61	1.77 ± 1.82	17; 0.001

AS = active sleep, ns = non-significant, QS = quiet sleep. Statistical data are indicated for Wilcoxon matched-pairs rank test DAN score baseline versus maximum DAN score post stimulation.

**Table 4:** DAN score changes according to SNR intensity and sleep state.

		DAN Score Baseline (Mean ± SD)	Maximum DAN Score Post Stimulation (Mean ± SD)	N; *p* Value
QS	10–50 lux	0.43 (±0.38)	0.89 (±0.44)	21; <0.001
	>50 lux	0.43 (±0.35)	0.89 (±0.69)	17; <0.01
AS	10–50 lux	0.80 (±0.82)	1.38 (±0.87)	10; <0.1
	>50 lux	-	-	-

AS = active sleep, QS = quiet sleep. Statistical data are indicated for Wilcoxon matched-pairs rank test DAN score baseline versus maximum DAN score post stimulation.

## Data Availability

The data presented in this study are available on request from the corresponding author.
